# EXPLORING THE IMPACT OF COGNITIVE DYSFUNCTION, FATIGUE, AND SHORTNESS OF BREATH ON ACTIVITIES OF DAILY LIFE AFTER COVID-19 INFECTION, UNTIL 1-YEAR FOLLOW-UP

**DOI:** 10.2340/jrm.v56.35403

**Published:** 2024-06-25

**Authors:** Ann BJÖRKDAHL, Marie GUSTAFSSON, Hilda ÖHLÉN, Sara JARL, Iolanda SANTOS TAVARES SILVA

**Affiliations:** 1 Sahlgrenska University Hospital, Occupational Therapy and Physiotherapy, Gothenburg; 2 University of Gothenburg, Sahlgrenska Academy, Institute of Neuroscience and Physiology, Gothenburg; 3 Primary Care Rehabilitation, Region of Västra Götaland, Gothenburg, Sweden

**Keywords:** daily activities, activity restrictions, post-COVID-19, experience, drowsiness, powerlessness, mental fatigue, cognitive dysfunction, long-term

## Abstract

**Objective:**

Despite expanding knowledge on COVID-19, the long-term effects on daily-life activities remain unclear. The prevalence and changes in fatigue, cognitive dysfunction, and activity limitations in the first year after COVID-19 infection in hospitalized and non-hospitalized patients were explored.

**Subjects:**

A total of 122 patients were recruited from hospital care and 90 from primary care.

**Method:**

Baseline data comprised the Montreal Cognitive Assessment and Trail Making Test. Participants were followed up at 3 and 12 months using these tests and a semi-structured interview to identify symptoms and how they affected participation in daily-life activities. Both within- and between-group analyses were performed to explore changes over time and compare groups.

**Result:**

High levels of fatigue and cognitive dysfunction were found in both groups, which persisted for 12 months. A significant impact on daily-life activities was also observed, with marginal change at the 12-month follow-up. The hospital care group performed worse than the primary care group in the cognitive tests, although the primary care group perceived a higher level of fatigue and cognitive dysfunction. Activity limitations were higher in the primary care group than in the hospital care group.

**Conclusion:**

These findings highlight the need for long-term follow-up and further investigation of the impact of persistent deficits on rehabilitation.

LAY ABSTRACT

Despite expanding knowledge on COVID-19, the long-term effects on daily-life activities remain unclear. This study explored the prevalence and changes in fatigue, cognition, and activity during the year following COVID-19 infection and compared hospitalized and non-hospitalized patients. In total, 122 patients were recruited from hospital care and 90 from primary care. The participants underwent 2 cognitive tests (Montreal Cognitive Assessment and Trail Making Test) and an interview regarding their experience of symptoms of fatigue and cognitive deficits and their impact on daily-life activities. Changes over 12 months were monitored and compared between groups. The findings showed high levels of fatigue and cognitive dysfunction and a significant impact on daily-life activities that persisted for 12 months in both groups. The hospital care group performed worse than the primary care group in the cognitive tests, although the primary care group experienced more cognitive problems, fatigue, and activity limitations.

It is now well established that COVID-19 infection involves multiple organs and systems, such as the respiratory, cardiovascular, neurological, gastrointestinal, endocrine, and musculoskeletal systems, which may impact in many different ways (1). Most patients with COVID-19 recover following mild symptoms, and only a subset are diagnosed with severe disease that requires hospital care (HC) (2, 3). Following hospitalization and intensive care, the disease appears to have a more dramatic course and less favourable long-term outcomes, especially for men. However, evidence suggests that even patients who are not in need of acute hospitalization can suffer from long-term persistent problems, which are more likely to affect women (3–5). Persistent symptoms are defined by the World Health Organization (WHO) as “post-COVID-19 condition” (PCC), which “occurs in individuals with a history of probable or confirmed SARS-CoV-2 infection, usually 3 months from the onset, with symptoms that last for at least 2 months and cannot be explained by an alternative diagnosis” (6). The prevalence of PCC varies considerably between reports, and a recent systematic review of 31 studies reported a prevalence of 9–81% across varied populations (7). A Swedish population-based study that comprised a near-complete dataset of inpatient, outpatient, and primary healthcare data found that 2% of all COVID-19 cases in the 2 largest regions in Sweden (4.1 million inhabitants) had a registered diagnosis of post-COVID-19 condition (PCC; International Classification of Diseases 10th Revision code U09.9) (5).

Significant differences in the development of PCC was shown (between patients requiring intensive care (36.9%) but no difference was shown between hospitalized patients not in need of intensive care and non-hospitalized patients (8.3% respectively) (5). PCC is more prevalent in females than in males (5, 8) and is not limited to older adults (9), and individuals with PCC have been shown to be more likely to have a higher level of education (5).

The most persistent symptoms reported are fatigue, shortness of breath, cognitive deficits, headache, myalgia, pain in the chest and joints, smell and taste dysfunction, cough, fever, insomnia, and cardiac and gastrointestinal issues (10–12). Fatigue is defined as a subjective feeling of tiredness and a lack of energy that significantly impacts an individual’s ability to perform activities of daily life (13); thus, the experience may differ depending on one’s demands and responsibilities. The prevalence of fatigue varies across settings and different time points depending on how it is measured (e.g., self-reported complaints versus using validated instruments). One systematic review of 41 studies evaluating the prevalence of fatigue in PCC recovery found that, irrespective of the setting or temporal characteristics, self-reported fatigue had a prevalence rate of 42% during the first 6 months of recovery (14). Across different settings, 30–60% of patients treated as either inpatients or outpatients report fatigue. In the general population, only 10% report experience of fatigue after a COVID-19 infection, whereas as many as 90% of individuals recruited via social media report fatigue (14).

The current literature on the connection between cognition and COVID-19 is limited, yet evidence from prior coronavirus epidemics and acute respiratory distress syndrome (ARDS) suggests that many people will experience functional cognitive and occupational performance deficits (15). After recovery from the acute phase of COVID-19 infection, neurological involvement seems to persist and may manifest as cognitive dysfunction (16). The mechanisms underlying cognitive impairment after COVID-19 are thought to involve long-term tissue damage and unresolved inflammation (10, 17) Frequently reported dysfunctions include impaired attention, memory, and executive functions (17, 18). Cognition is essential for effective performance across the broad range of activities of daily life such as work, educational pursuits, home management, and leisure (19, 20). There is an interaction between a person’s cognitive functioning and factors such as context, environment, and activity demands (19), which is essential to take into account when trying to understand the impact of PCC on activities of daily life.

Shortness of breath is the second most common complaint, with a pooled estimate of 35% of cases having dyspnoea, which significantly hinders the performance of physical activities (21). Reduced diffusion capacity was the major respiratory impairment 3–6 months following COVID-19, with hospitalization as the most important risk factor (22).

As COVID-19 is a new disease, it may take years to characterize the exact nature and temporal extent of the long-term neurocognitive sequelae and the consequences for activities of daily life. Thereby systematic longitudinal follow-up will be required (23). Although knowledge of the persistent symptoms following COVID-19 infection is improving, few studies have gained an in-depth understanding of the implications of the various symptoms and their effect on everyday life. We aimed to better understand the limitations in daily activity attributed to fatigue, cognitive dysfunction, and breathlessness and examine the improvements made during the year following the illness. Because the study included two cohorts (i.e., hospitalized and non-hospitalized patients), we also explored the differences and similarities in symptoms depending on the type of acute illness.

## Research questions

- How do cognitive dysfunction, fatigue and breath-lessness after COVID-19 infection affect activities of daily living?
- What is the prevalence of fatigue and cognitive dysfunction over time for hospitalized and non-hospitalized COVID-19 patients, and how does it change over the year following the infection?
- Are there differences in cognitive dysfunction, fatigue, breathlessness or impact on activities of daily life depending on whether you have been hospitalized or not?

## Methods

The study was part of a prospective 1-year follow-up study in hospitalized (i.e., HC) and non-hospitalized (i.e., primary health care [PC]) COVID-19 patients examining fatigue, cognitive function, and participation in activities of daily life (FOU I Sverige Dnr 274943, https://www.researchweb.org/is/sverige/project/274943). The focus of the study was the manifestation of symptoms and their impact on everyday life. The study was approved by the Swedish Ethical Review Authority (Dnr: 2020-03222) and complied with the Declaration of Helsinki. Written informed consent was obtained from all subjects involved in the study.

### Materials

The study included 122 patients admitted for HC after being infected with SARS-CoV-2 (between 1 July 2020, and 28 February 2021) and 90 patients who were enrolled in PC rehabilitation visiting an occupational therapist (between 1 September 2020, and 31 August 2021) because of PCC-related rehabilitation needs.

The eligibility criterion for HC patients was admission to Sahlgrenska University Hospital (SU) with a hospital stay of ≥ 5 days in total or following intensive care. The eligibility criterion for the PC group was patients seeking rehabilitation following COVID-19 infection admitted to PC rehabilitation units in Gothenburg, Sweden. The time from onset of COVID-19 infection to enrolment in PC ranged from 2–56 weeks (50% within 16 weeks, 10% over 40 weeks) because many patients believed that their symptoms would resolve on their own or were not certain how an occupational therapist in PC rehabilitation could help them. Follow-ups were conducted at 3 and 12 months after enrolment. In the PC group, 13 patients had been admitted to hospital due to COVID-19 before inclusion in the study. However, admission for these patients was before or after the recruitment period for the HC group. Therefore, these 13 patients were classified into a third group, PC+, and were not combined with those in the PC group who did not require HC.

Exclusion criteria for all patients were: the inability to understand and participate in an interview on symptoms and their impact on daily activities, cognitive or physical inability to perform cognitive screening, and unable to live independently before the onset of COVID-19 infection.

Recruitment of hospital patients was coordinated with another study with similar objectives being conducted simultaneously (“Life in the time of Covid study in Gothenburg” [GOT-LOCO]) (24). A study coordinator identified eligible participants at different units within the hospital. A local occupational therapist (OT) at the unit approached and informed patients concerning the study. After obtaining informed consent from the patient, the OT collected data as a first assessment. All OTs involved in the study underwent training for the test procedure before recruitment began. The clinical OT in PC informed and invited all new COVID-19 patients to participate in the study at enrolment in PC. The test procedure in PC was identical to that used by the OT at the hospital. At the 3- and 12-month follow-ups, patients were evaluated and interviewed by independent evaluators.

### Data collection

Baseline data collection was undertaken at the clinic where the patient received treatment (i.e., hospital or PC clinic) and consisted of cognitive screening using the Montreal Cognitive Assessment (MoCA) and Trail Making Test A and B (TMT). Descriptive data, such as date of COVID-19 infection onset, length of hospital stay, and intensive care, were retrieved from medical records.

Follow-up data collection was performed 3 and 12 months after the baseline assessment. Patients visited the occupational therapy and physiotherapy department at SU and met with the OT. The visit lasted approximately 1.5 hours, and the same tests as the first assessment were administered (i.e., the MoCA and TMT), with the addition of a self-estimation of fatigue using a visual analogue scale (VAS) ranging from 0–100. A semi-structured interview was conducted to identify persisting symptoms of impaired cognition, fatigue, and breathing difficulties, and how these symptoms impacted on everyday life. The interview lasted approximately 1 hour, and patients were asked to describe how they were affected in daily-life activities and if they could explain what caused the eventual problems, such as lack of energy, difficulty finding words, or staying focused. Patients’ answers were subsequently transcribed. After we had conducted interviews with several patients, we noted that fatigue was being reported in 3 forms. Therefore, the questions in the interview guide were adjusted to specifically ask patients about these 3 aspects of fatigue: sleepiness, powerlessness, and mental fatigue. The presence or absence of the different symptoms of fatigue and cognitive dysfunction was confirmed and described as proportions in the HC and PC groups. Based on participants’ descriptions of activity limitations in the domains of personal care (personal activities of daily life [PADL]), household (instrumental activities of daily life [IADL]), leisure, and work or study, the OT rated the impact as no impact = 0, moderate impact = 1 and great impact = 2.

After the initial rehabilitation in hospital (HC) and in primary care occupational therapy (PC), both groups may have sought and received further rehabilitation in primary care during the follow-up year if needed.

### Instruments

The MoCA is a brief, sensitive, cognitive screening tool that assesses several cognitive domains. The maximum score is 30, and a score below 26 points indicates a need for further investigation. The MoCA has high reliability and validity and is more sensitive for detecting cognitive deficits than many other screening tools (25). Parallel versions were used at the different assessments.

The TMT is a widely used neuropsychological test that evaluates 2 basic processes: visual search and visuomotor speed. TMT B is a more difficult task than TMT A because of the need to alternate between numbers and letters, and involves working memory and cognitive flexibility (26). The score is the time for completion, in seconds.

### Data analysis

We calculated descriptive statistics for all 3 groups. Comparisons between the HC and PC groups were performed using parametric (Student’s *t*-test) and non-parametric tests (Mann–Whitney *U* test and χ^2^ test). Because of the small sample size, the PC+ group was not included in the comparisons. The experience of presence or absence of different types of tiredness and cognitive dysfunction is presented descriptively as proportions and was analysed statistically with a χ^2^ test both within group and between groups at 3- and 12-month assessments. The within-group changes in the MoCA and TMT scores were analysed using paired samples *t*-tests. Because there were differences between the groups in age, sex, and education, adjustments were made by calculating the *t*-score for the TMT B score (27) and z-values for the MoCA scores (28). The performance of TMT A is a cognitively less demanding task than that of TMT B and thus was not used for comparisons with *t*-score calculations. For the *t*-score the mean norm score is T50 and 1 SD is ± 10 and similarly the mean norm z-score is 0 and 1 SD is ± 1, which was used in the interpretation of the result. The ratings of activity limitations (0–2) were described as proportions in each category for each of the 4 activity domains. Comparisons between patients with severe acute disease (i.e., HC) and those with milder acute disease (i.e., PC) were performed with the caveat that they were not directly comparable because of differences in time from onset to assessment. Paired sample analyses for these variables were made by Wilcoxon signed rank test.

## RESULTS

The sample consisted of 3 groups: HC (*N* = 122), PC (*n* = 77) and PC+ (*n* = 13). Table I shows the age, sex, and education of the 3 groups. Days in hospital are provided for the HC and PC+ groups. Time to first assessment in the 2 PC groups varied considerably: PC had a median of 16.5 (2–56) weeks post-infection, and PC+ had a median of 17 (4–30) weeks post-infection. In PC, 40% had their first assessment within 3 months, 35% within 6 months. In PC+ all participants were assessed for the first time within 6 months.

**Table I:** Descriptive data of the sample

Variables		Hospital care (HC)	Primary care (PC)	Primary care with hospital days (PC+)

		*n* = 122	*n* = 77	*n* = 13
Age	Mean (SD) Median (range)	63.94 (13.18) 65 (20–93)	48.90 (13.31) 48 (16–75)	56.15 (8.4) 56 (42–66)
Sex, %	Male/female	73.0/27.0	31.2/68.8	61.5/38.5
Education, %	> 12 years	44.5	75.3	69.2
Hospital care (days)	Mean (SD) Median (range)	37 (40) 20 (5–200)	–	18 (11) 14 (6–42)
Time to first assessment (weeks)	Median (range)	–	16.5 (2–56)	17.0 (4–30)

SD: standard deviation.

**Fig. 1:**
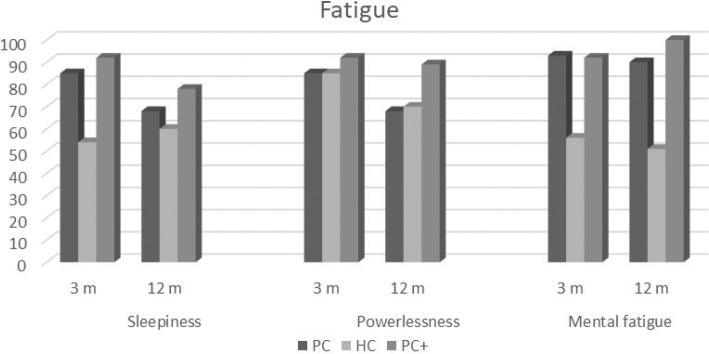
**Percentage of patients who experienced different kinds of fatigue in each group.** Fatigue was described as sleepiness, powerlessness, and mental fatigue. PC: primary care; HC: hospital care; PC+: PC plus HC.

Fatigue was common after COVID-19 infection at both the 3- and 12-month follow-ups. On the VAS, the PC and PC+ groups both had a mean score of 56 (standard deviation [SD] 23.5 and 25.6, respectively), and the HC group scored 48 (SD 25.5) at 3 months. At 12 months, the score remained high in all 3 groups: PC: 54 (SD 25.0), HC: 42 (SD 25.5) and PC+: 62 (SD 26.3). There were significant differences in fatigue between PC and HC at both follow-ups (*p* = 0.03 [confidence interval (CI) 0.862–16.702] and *p* = 0.01 [CI 2.946–21.445]), where the PC group experienced greater fatigue than the HC group. The preliminary analysis of the first 10 interviews revealed that tiredness manifested in various ways. Therefore, fatigue was categorzed into 3 forms: sleepiness, powerlessness, and mental fatigue. The presence of all 3 types of fatigue was high in all 3 groups: sleepiness (56%–92%), powerlessness (85%–92%), and mental fatigue (58%–93%; Fig. 1). However, the HC group had significantly lower levels of sleepiness and mental fatigue than PC at 3 months (HC versus PC: *p* < 0.001 for both sleepiness and mental fatigue; Fig. 1). At 12 months, sleepiness decreased in PC and PC+ but increased slightly in HC, which is why there were no longer significant differences between PC and HC. However, mental fatigue was still significantly different between the HC group and the PC group (*p* < 0.001) (Table II). Almost half of all subjects reported sustained respiratory impairment causing breathlessness at 3 months (44.2% of PC, 38.5% of PC+, and 35.5% of HC), and at 12 months, the number with breathing problems decreased slightly for the PC group (28.6%, *p* = 0.005) but remained largely unchanged for the HC group (37.7%, *p* = 0.258). The PC+ showed a lower percentage of breathing problems at 12 months (23.1%), but the difference was not analysed due to the small sample size (22).

**Table II:** χ^2^ comparisons of experience of fatigue and cognitive dysfunction between primary care (PC) and hospital care (HC)

Variables	3 months			12 months		

Comparisons PC–HC	χ^2^	*p*-value	phi	χ^2^	*p*-value	phi
Sleepiness	15.416	**< 0.001**	–0.325	1.177	0.278	–0.108
Powerlessness	0.000	1.000	–0.005	0.000	1.000	0.005
Mental fatigue	25.696	**< 0.001**	–0.402	18.344	**< 0.001**	–0.385
Breathing problem	0.077	0.908	–0.022	2.394	0.122	0.149
Cognitive dysfunction	15.447	**0.001**	–0.320	9.615	**0.002**	–0.279

Within group comparison 3 and 12 months	PC			HC		

	χ^2^	*p*-value	phi	χ^2^	*p*-value	phi

Sleepiness	4.598	**0.032**	0.322	5.509	**0.019**	0.310
Powerlessness	2.962	0.085	0.269	6.806	**0.009**	0.356
Mental fatigue	0.045	0.832	0.139	6.151	**0.013**	0.336
Breathing problem	7.815	**0.005**	0.387	1.281	0.258	0.171
Cognitive dysfunction	1.880	0.170	0.266	9.383	**0.002**	0.388

At the 3- and 12-month follow-ups, a considerable proportion of participants continued to experience cognitive dysfunction. In the PC group, 92% at 3 months and 84% at 12 months (*p* = 0.170) reported that they had cognitive problems. All participants in the PC+ group reported cognitive problems at both time points, and 64% and 60% of the HC group reported cognitive dysfunction at 3 and 12 months, respectively (*p* = 0.827). Results of the MoCA and TMT B are presented in Table III. The proportion scoring > 1 SD below norm on MoCA z-score was between 51% and 31% in the PC, 52% and 48% in HC and 50% and 30% in PC+ on the 3 assessments, while the MoCA raw sum scores were above cut-off in all 3 groups at all assessments except for the first assessment in HC.

**Table III:** Median scores and range of Montreal Cognitive Assessment (MoCA) and Trail Making Test (TMT) scores in the 3 groups at baseline, 3 months, and 12 months. Comparisons between primary care (PC) and hospital care (HC)

Time	PC			HC			PC+			Sign *p* < 0.05		Cohen’s d
MoCA	Sum score, median (range)	z-score, mean (SD)	% > 1 SD below norm	Sum score, median (range)	z-score mean (SD)	% > 1 SD below norm	Sum score, median (range)	z-score mean (SD)	% > 1 SD below norm	Comparison PC and HC MoCA	Comparison PC and HC MoCA z-score	Effect size z-score comparisons
Baseline	27 (19–30)	−1.1 (1.1)	51%	25 (8–30)	−1.7 (1.9)	52%	26 (15–29)	−1.5 (1.9)	38%	*p* < 0.001 (CI 1.82–4.02)	*p* = 0.028 (CI 0.06–1.05)	0.770 (CI 0.470–1.068)
3 months	28 (22–30)	−0.8 (1.0)	33%	26 (11–30)	−1.2 (1.8)	48%	28 (16–30)	−1.1 (2.1)	46%	*p* < 0.001 (CI 1.45–3.57)		
1 year	28 (18–30)	−1.0 (1.1)	36%	26 (14–30)	−1.0 (1.6)	49%	26 (22–28)	−1.2 (1.2)	50%	*p* = 0.002 (CI 0.70–2.98)		
TMT B	Seconds, mean (SD)	T-value (SD)	% > 1 SD below norm	Seconds, mean (SD)	T-value (SD)	% > 1 SD below norm	Seconds, mean (SD)	T-value (SD)	% > 1 SD below norm	Comparison PC and HC TMT	Comparison PC and HC TMT *t*-score	Effect size *t*-score comparison, Cohen’s d
Baseline	80 (47)	49 (16)	16%	142 (85)	41 (15)	41%	109 (77)	40 (27)	38%	*p* < 0.001 (CI −84.1 to −40.6)	*p* = 0.001 (CI 3.2–12.6)	0.551 (CI 0.20–0.82)
3 months	71 (48)	54 (15)	13%	107 (67)	48 (15)	21%	82 (56)	49 (22)	15%	*p* < 0.001 (CI −55.7 to −16.7)	*p* = 0.03 (CI 0.39–10.0)	0.351 (CI 0.03–0.68)
1 year	59 (37)	58 (14)	6%	94 (64)	53 (14)	15%	85 (62)	50 (25)	25%	*p* < 0.001 (CI −55.7 to −15.1)		
TMT A												
Baseline	34 (15)			56 (36)			53 (42)			*p* < 0.001 (CI −31.0 to −13.6)		
3 months	30 (18)			41 (24)			52 (55)			*p* = 0.004 (CI −17.4–3.4)		
1 year	28 (13)			40 (33)			33 (22)			*p* = 0.012 (CI −22.6 to −2.8)		

The calculated mean z-score and standard deviation (SD) in all groups and all assessments are presented together with the percentage of the groups that at each time point scored below 1 SD of the normative mean (z = 0). Similarly, the Trail Making Test (TMT) B mean time in seconds and T-value with SD are shown, together with the percentage of the groups with a T-value 1 SD below the normative average (T50). For the z-score, 1 SD is indicated by a score < −1, and for the T-value, a score below T40 is > 1 SD. For TMT A, only raw scores are presented. Comparisons between PC and HC groups of MoCA and TMT B scores, raw scores, z-scores and *t*-scores, together with effect sizes (Cohen’s d), are provided in the right-hand column.

CI: 95% confidence interval.

In the comparisons between groups on TMT B, the PC group performed significantly better than the HC group at first assessment and at 3 months even after adjusting for age, sex, and education. The effect size was small to moderate. There were significant differences between the PC and HC groups in the raw scores of both TMT A and B, across all assessments (Table III).

MoCA scores differed significantly between the PC and HC groups for all 3 assessments when age, sex, and education were not adjusted; however, when adjusted for age, sex, and education, a significant difference was observed at first assessment only. There were similar proportions of participants in PC and HC with a score of more than 1 SD below the mean norm score (Table III), and the analyses revealed a decrease between baseline and 3 months but the proportions remained almost the same between 3 and 12 months. For TMT B, the proportion of participants scoring below the mean norm score was lower in the PC group than in the HC group.

**Fig. 2:**
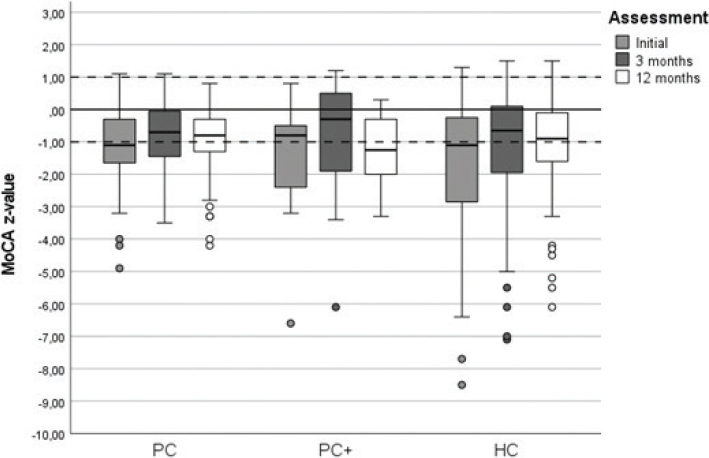
**Boxplot showing z-scores of the Montreal Cognitive Assessment (MoCA) at the first assessment and 3- and 12-month follow-up for the 3 groups: primary care (PC), hospital care (HC), PC plus HC (PC+).** The solid line at 0 represents population mean and the dashed lines indicate ± 1 SD, the normal range.

**Fig. 3:**
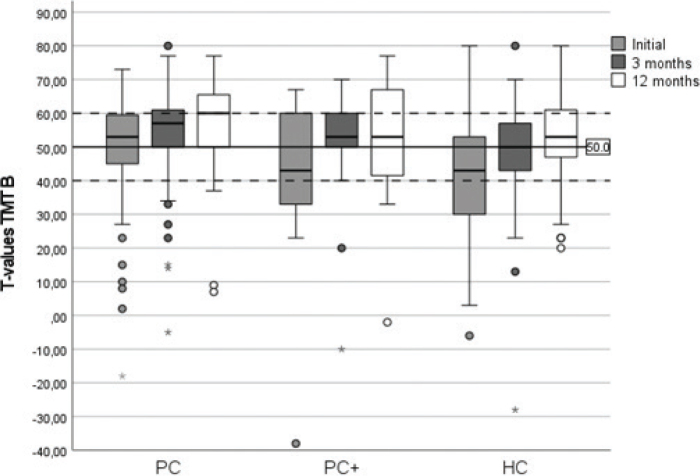
**Boxplot showing *t*-scores of the Trail Making Test (TMT) B at the first assessment and the 3- and 12-month follow-up for the 3 groups: primary care (PC), PC and earlier hospital care (PC+), and hospital care (HC).** The solid line at T50 represents the population mean and the dashed lines indicate ± 1 SD, the normal range.

Results for the MoCA and TMT B and the score change over time are shown as boxplots for each group in Figs 2 and 3. Significant positive changes in the TMT B *t*-score were found in all 3 groups at all assessments except for the PC+ group between 3 and 12 months (Table IV). The PC group showed a significant change in MoCA z-score between baseline and 3 months as well as between 3 and 12 months. The HC group showed a significant change in MoCA z-score from baseline to 3 months only, and the PC+ group did not show a significant positive change in MoCA at any timepoint. The magnitude of significant changes in the MoCA z-scores between assessments was smaller than that for TMT B scores.

**Table IV:** Paired comparison of Trail Making Test (TMT) B and Montreal Cognitive Assessment (MoCA) scores between the 3 groups

Test	Baseline–3 months			3–12 months			Baseline–12 months		
	Sign *p* < 0.05	95 % CI	Effect size, Cohen’s d	Sign *p* < 0.05	95 % CI	Effect size, Cohen’s d	Sign *p* < 0.05	95 % CI	Effect size, Cohen’s d
MoCA z-score									
PC	0.010	–0.596 to –0.0842	–0.324	0.020	0.052–0.581	0.330	No sign	–0.310–0.265	
HC	0.023	–0.712–0.054	–0.428	No sign	–0.217–0.400		No sign	–0.736–0.122	
PC+	No sign	–1.125− 0.356		No sign	–1.308–1.188		No sign	–1.372–0.992	
TMT *t*-score									
PC	0.002	–8.294 to –2.001	–0.419	0.022	–6.042 − –0.503	–0.414	< 0.001	–4.702 to –5.123	–0.747
HC	< 0.001	–8.738 to –2.577	–0.428	0.037	–7.611 − –0.237	–0.262	< 0.001	–15.279 to –7.636	–0.781
PC+	0.015	–15.490 to –2.048	–0.788	no sign	–6.517–5.767		0.021	–24.898 to –2.048	–1.052

SD: standard deviation; CI: 95% confidence interval; PC: primary care; HC: hospital care.

**Fig. 4:**
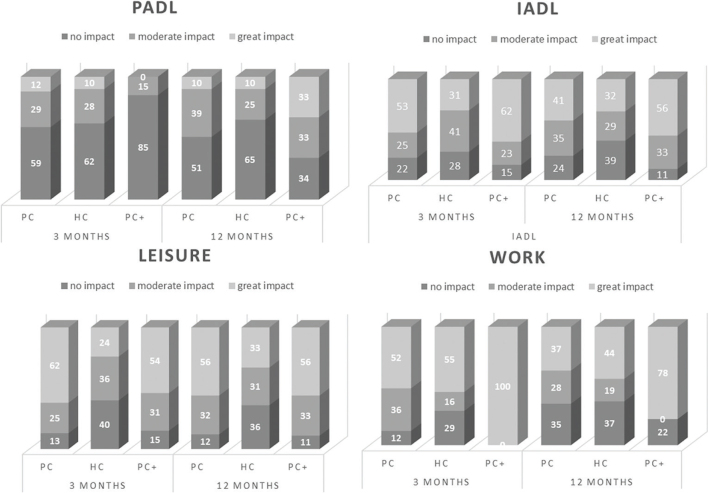
**Diagrams showing rated difficulties in performance of the activities personal care (PADL), instrumental activities of daily life (IADL), leisure, and work in the 3 groups PC, PC+, and HC at 3- and 12-month follow-up.** Bars show the proportions (%) of problems (no, moderate and great) rated for the 4 activities, for each group, and follow-up time.

The effect of symptoms on everyday activities participants experienced and conveyed to the OT during the interviews at 3 and 12 months are shown in Fig. 4. Each activity domain is presented separately: PADL, IADL, leisure, and work. Fig. 4 shows that for all activities except for PADL, more than 60% of participants experienced moderate to extensive activity limitations at 3 months, and these persisted at a similar level at 12 months. Return to work (RTW) was slow. Of the participants who were of working age (i.e., excluding those who were retired or unemployed), 45% had fully returned and 27% had not returned to work at all in the PC group (*n* = 46), at 3 months. In the HC group (*n* = 44), 24% had fully returned and 58% had not returned to work. In the PC+ group (*n* = 7), 15.5% had fully returned to work and 38.5% had not returned to work. At 12 months, RTW was as follows: PC: 39% full RTW and 41% no RTW, HC: 54% full RTW and 32% no RTW, and PC+: 14% full RTW and 43% no RTW.

Participants reported during the interviews that they experienced difficulties in completing an activity, in whole or in part, because of a lack of energy or shortness of breath. Activities were also hindered because of brain overload due to mental fatigue. Cognitive functioning worsened when participants were tired; however, they also reported specific cognitive difficulties when they were more alert. The cognitive dysfunctions affecting daily activities primarily involved attention, working memory, and speed of information processing (e.g., forgetting appointments, maintaining focus, and keeping track of the activity being undertaken). Participants also experienced problems in understanding and remembering instructions and responding appropriately.

## DISCUSSION

Through interviews and assessments of 212 patients who were either hospitalized with severe disease or received PC with milder acute COVID-19 disease, our study revealed that extensive symptoms persisted at both 3 and 12 months after infection. Fatigue was commonly experienced at both follow-up periods, with only small changes between assessments. However, the degree of fatigue was significantly higher in the PC group than in the HC group. Additionally, cognitive problems were typical across all groups and persisted until the 12-month follow-up. Although self-rated cognitive complaints were higher in the PC groups than in the HC group, the HC group scored worse than both PC groups on the cognitive assessments. Based on participants’ accounts, it was evident that the symptoms following COVID-19 infection greatly impacted on activities of daily life; indeed, few individuals had returned to work at 12 months.

A study conducted in Sweden reported white-matter lesions on brain magnetic resonance imaging following COVID-19 infection, which may explain the neuropsychological deficiencies; moreover, they highlighted the importance of thoroughly examining patients who are experiencing cognitive problems, regardless of disease severity (18). Indeed, there is mounting evidence for neural damage and impaired neurotransmission following COVID-19 infection, which are manifested as both impaired cognition and fatigue (29-31).

Our finding of lower MoCA and TMT B scores in the HC group than in the PC groups is in line with other studies reporting that hospitalized patients perform worse than non-hospitalized patients in processing speed and the MoCA (32, 33). However, they also found that, using more detailed neuropsychological tests, non-hospitalized patients experience similar levels of cognitive impairment to hospitalized patients across most domains. When we adjusted for age, sex, and education, we found a significant difference only in the MoCA score at baseline, which may be attributed to the difference in time to assessment, where the HC group would have been assessed closest to COVID-19 infection onset.

Similar to our findings, the study by Mirfazeli et al. (34) and others (33) showed no relationship between MoCA score at follow-up and disease severity. It is worth noting that although the MoCA is a valid, reliable, and sensitive tool for assessing mild cognitive dysfunction (35, 36), it is first and foremost a screening tool for global cognitive function. Therefore, the MoCA cannot be used to evaluate an individual’s capacity to perform complex activities. Such activities are dependent on interactions between the individual, the environment, and task demands (20). By contrast, during the interviews, participants’ responses to questions concerning their cognitive deficits were related to daily-life experiences, which may explain the higher degree of perceived cognitive impact in the PC group, despite performing better than the HC on the tests. Because a larger proportion of individuals in the PC group had a higher education and were of working age, it is also reasonable to assume that this group would have higher cognitive demands in daily life.

The information gathered from interviews confirmed previous findings regarding the cognitive domains affected by COVID-19 infection (30–32). Participants reported experiencing cognitive problems involving attentional processes, such as noticing, listening, taking in information, and maintaining focus over time. Memory, especially working memory, was also affected, which resulted in a need to repeat and write everything down. This led to learning difficulties, problems with responsibilities at work, and carrying out daily activities. For many, these problems contributed to difficulties in understanding and processing complex information, and their cognitive dysfunctions had a significant negative impact on participants’ ability to fulfil their previous occupational roles. Although previous studies have demonstrated only limited cognitive dysfunction following COVID-19 infection, patients report decreased cognitive function alongside fatigue, which considerably hinders their ability to work and be as active as they were before the infection (37). This was confirmed by the difficulty ratings for performance in the activity domains of IADL, leisure, and work from the present study.

In the present study, more than 80% of participants described experiencing persistent fatigue. The incidence of fatigue has been shown to vary depending on how it is measured (14). In our study, participants were asked about their fatigue during the interview, which allowed for extensive descriptions of the limitations to activities due to fatigue; this may have led to a high prevalence of fatigue. We assessed fatigue using the VAS at both 3- and 12-month follow-up. The perception of fatigue was, similar to the perception of cognitive dysfunction, higher in the PC group than in the HC group. However, it is worth noting that two-thirds of the PC group were women, whereas only one-third of the HC group were women, and it has been shown that the prevalence of PCC fatigue is significantly higher among women (13).

The participants described 3 types of fatigue: sleepiness, powerlessness, and mental fatigue. All forms significantly impacted daily-life activities. Clinical fatigue has been described in other diagnoses as comprising 3 components: “(1) generalised weakness, resulting in an inability to initiate certain activities; (2) easily fatigued and reduced capacity to maintain performance; and (3) mental fatigue, resulting in impaired concentration, loss of memory and emotional lability,” each having a different impact on daily life (38). These descriptions are consistent with the descriptions in the present study of powerlessness (1 and 2) and mental fatigue (3) in our study. The HC group reported that their fatigue was primarily associated with powerlessness, especially at 3 months. Because a significant proportion of people in this group had been severely ill and inactive for several weeks, they had reduced muscle strength and breathing difficulties, even at 3 months, which may explain their lack of energy and stamina. This may have improved over time through physical training and recovery. Participants in the PC+ group had also been previously hospitalized but were subsequently admitted to PC for rehabilitation because of insufficient improvement, which may explain why this group had a greater lack of energy and more residual problems than the other 2 groups. In the PC and PC+ groups, mental fatigue was the most problematic form of fatigue. Furthermore, we found that the PC group had significantly more problems with fatigue than the HC group, which could not be attributed to time to assessment. To date, a significant association between severity in the acute phase and subsequent persistent fatigue syndrome has not been reported (33, 34).

Experience of breathlessness, which was high in all groups, also contributed to the feeling of powerlessness. Findings show that, predominantly, hospitalized male patients experience breathlessness because of low diffusion capacity after COVID-19 (22). How-ever, many women presented with mild COVID-19 in the acute phase, experience a higher risk of increased breathlessness at 3–6 months, possibly due to a patho-logical heart-rate response or oxygen desaturation. The findings suggest that these physiological signs represent different phenotypic disease entities (22). This could be an explanation for similar findings in both PC and HC at follow-up.

The interviews at the 3- and 12-month follow-ups were thorough and in-depth, which enabled us to gather examples of tasks that participants found difficult and understand why they found carrying out the activities challenging. It was revealed that fatigue, breathlessness, and cognitive deficits had an impact on participants’ ability to perform activities. Improvements in cognition and fatigue over time correlated strongly with the change in the ability to resume activities. A 2-year follow-up study conducted in Sweden found that, similar to our study, over 80% of patients had persistent cognitive deficits and fatigue following COVID-19 infection, and limitations in participation in daily activities, which had an effect on everyday life (39). Another study that compared pre- and post-COVID-19 infection status questioned whether minor functional limitations (based on functional assessments) following illness accurately reflect functional status, given that 56% of the study population was on sick leave, and 94% required rehabilitation following infection (40). Comparable to our findings regarding return to work, Van Wambeke et al. (41) found that 2 years after covid-19 infection, 36% of the participants had still not returned at all to work and only 40% had fully returned.

Participants stated in the interviews that they experienced difficulties in completing an activity in whole or in part because of a lack of energy, shortness of breath, mental fatigue, or cognitive impairment. A cognitive function that was particularly impaired following COVID-19 infection was attention, which is a fundamental cognitive process and a subsystem for other cognitive functions and thus is crucial for managing everyday life (31). Because of the cognitive deficits, participants experienced problems in remembering appointments, staying focused, and processing and understanding complex information, which are processes essential for carrying out daily activities and work.

Fatigue also significantly impacted on participants’ performance in activities, family, work, and lifestyle, which highlights the importance of providing rehabilitation for fatigue (42). A study conducted in Switzerland explored the effectiveness of energy management education (EME) treatment for COVID-19 patients. The focus for EME is reflections on “energy account,” “break management,” “occupational balance,” and “effective communication.” They reported that the primary challenge for occupational therapists was the short duration of fatigue experienced by patients because of the sudden onset of COVID-19 infection, which resulted in a discrepancy between self-concept, self-perception, and performance. Because little is known about the recovery from the consequences of COVID-19 infection, patients felt insecure, fearful, and anxious regarding their recovery (42); these feelings were also experienced by the patients in our study. Based on the results of our study and previous studies, there is a need for interventions to support individuals experiencing performance problems to enable them to resume everyday activities and RTW long-term (15).

### Strengths and limitations

An important limitation and a possible reason for the initial group differences is the difference in time to first assessment between groups. On one hand, the PC group had a longer time to recover, which may have resulted in better performance in the assessments. On the other hand, they may have been a select group of individuals who had significant persistent problems in need of rehabilitation. However, the main point of the group comparison was to show that significant problems also exist in the non-hospitalized group and that these persist in the long term. Although group affiliation could be revealed during the interviews and assessments, which could have influenced evaluators’ activity limitation ratings, such bias is unlikely because the interviews were conducted by different assessors and the sample size was substantial. Despite the aforementioned limitation, a strength of the study is the comparison of outcomes between a hospitalized group and a non-hospitalized group that underwent the same assessments. Cognitive dysfunctions were revealed to be a significant problem for the participants. One limitation is that we administered only a screening instrument (i.e., the MoCA), instead of a complete neuropsychological test battery. Similar to other studies, cognitive complaints could not be verified in the present study using raw scores of MoCA as many of the participants scored above the cut-off (43, 44). Cognitive tests are known to be affected by age, sex, and educational level. However, we tried to control for this weakness by adjusting the scores for age, education, and sex using z-scores, which differed significantly between groups.

### Conclusion

A significant proportion of COVID-19 patients experienced persistent symptoms 12 months following illness, irrespective of the severity of the initial disease. Self-reported fatigue and cognitive complaints were significantly greater in the PC group than in the HC group. Improvements over time were small, and even at 12 months all participants continued to experience considerable limitations in participation in activities of daily life. Further research is needed on long-term outcomes and effective rehabilitation approaches.
